# Circulating immunophenotypes are potentially prognostic in follicular cell-derived thyroid cancer

**DOI:** 10.3389/fimmu.2023.1325343

**Published:** 2024-01-03

**Authors:** Anupam Kotwal, Michael P. Gustafson, Svetlana Bornschlegl, Allan B. Dietz, Danae Delivanis, Mabel Ryder

**Affiliations:** ^1^ Division of Diabetes, Endocrinology and Metabolism, University of Nebraska Medical Center, Omaha, NE, United States; ^2^ Division of Endocrinology, Diabetes, Metabolism, and Nutrition, Mayo Clinic, Rochester, MN, United States; ^3^ Divisions of Experimental Pathology and Transfusion Medicine, Mayo Clinic, Rochester, MN, United States; ^4^ Division of Laboratory Medicine, Department of Laboratory Medicine and Pathology, Mayo Clinic Arizona, Phoenix, AZ, United States; ^5^ Department of Laboratory Medicine and Pathology, Mayo Clinic, Rochester, MN, United States; ^6^ Department of Immunology, Mayo Clinic, Rochester, MN, United States; ^7^ Division of Medical Oncology, Mayo Clinic, Rochester, MN, United States

**Keywords:** immune cell, flow cytometry, prognosis, thyroid carcinoma, T cell, immune markers

## Abstract

**Background:**

Exploring the immune interface of follicular cell-derived thyroid cancer has prognostic and therapeutic potential. The available literature is lacking for comprehensive immunophenotyping in relation to clinical outcomes. In this study, we identify circulating immunophenotypes associated with thyroid cancer prognosis.

**Methods:**

We conducted a pilot observational study of adults with follicular cell-derived thyroid cancer who underwent surgery at our tertiary care referral center and had consented for flow cytometry on peripheral blood collected at the time of thyroidectomy.

**Results:**

Of the 32 included subjects, 20 (62%) had well differentiated, 5 (16%) had poorly differentiated, and 7 (22%) had anaplastic thyroid cancer. The most frequent AJCC stage was 4 (59%) and the ATA risk of recurrence category was high (56%). Patients with AJCC stage 3/4 demonstrated fewer circulating mononuclear cells (CD45+), more monocytes (CD14+), fewer total lymphocytes (CD14-), fewer T cells (CD3+), fewer CD4+ T cells, fewer gamma-delta T cells, fewer natural killer (NK) T-like cells, more myeloid-derived suppressor cells (MDSCs; Lin-CD33+HLADR-), and more effector memory T cells but similar CD8+ T cells compared to stage1/2. Immunophenotype comparisons by ATA risk stratification and course of thyroid cancer were comparable to those observed for stage, except for significant differences in memory T cell subtypes. The median follow-up was 58 months.

**Conclusions:**

Aggressive follicular cell-derived thyroid cancer either at presentation or during follow-up is associated with down-regulation of the T cell populations specifically CD4+ T cells, gamma-delta T cells, and NK T-like cells but up-regulation of MDSCs and altered memory T cells. These immunophenotypes are potential prognostic biomarkers supporting future investigation for developing targeted immunotherapies against advanced thyroid cancer.

## Background

The incidence of thyroid cancer has been steadily increasing. Follicular cell-derived differentiated thyroid cancers have a favorable prognosis with conventional treatment including thyroidectomy with or without radioactive iodine. However, at least 10% of patients develop radioactive iodine-refractory metastases to lung, bone, and other sites. In such cases, 5-year survival can be a dismal 15.3% (1). While aggressive multi-modality approaches and tyrosine kinase inhibitors have demonstrated improved outcomes, such therapies have toxicities and usually partial responses (2, 3). This has led to the exploration of immunotherapies for advanced thyroid cancer and stimulated an interest in understanding the effect of the immune system on thyroid cancer. The ligand for immune checkpoint programmed cell death protein 1 (PD-1) has been demonstrated on malignant thyroid cells (4, 5), thus leading to the trial of PD-1 inhibitors in anaplastic thyroid cancer (6). However, the effect of these therapies on advanced thyroid cancer has been unpredictable or poor (7). This could be due to the resurgence of an immunosuppressive tumor microenvironment as shown in a murine model of thyroid cancer (8).

The association between immune-mediated inflammation and follicular cell-derived thyroid cancer has been reported (9) as evidenced by a mixture of cytokines, chemokines, and immune cells in the tumor microenvironment. While an association between autoimmune thyroid disease and thyroid cancer has been reported in a database study (10), the impact of chronic lymphocytic thyroiditis on thyroid cancer prognosis remains unclear (11–14). To address this issue, studies have identified the increased immune suppressor cells, regulatory T cells (Tregs) (15), PD1+ T cells (15), myeloid-derived suppressor cells (MDSCs) (16), in circulation, and infiltrating the tumor of pathologically aggressive differentiated thyroid cancer (5, 17–19), while the association with effector CD8+ T cells has been mixed (20, 21). However, comprehensive examination of the relationship between circulating immunophenotypes and clinicopathologic outcomes in thyroid cancer patients remains limited. Our group has previously identified circulating immune cell profiles in thyroiditis caused by immune checkpoint inhibitors (ICIs) (22), as well as healthy and other malignancy patients (23, 24); and more immune activator T cell subpopulations in the thyroid glands of patients developing PD-1/PD-L1 inhibitor-induced thyroiditis (25). These studies highlight our ability to comprehensively analyze immunophenotypes in relation to clinically key factors. Based on the available literature and our previous work, we hypothesized that the circulating leukocyte populations, specifically suppressor (Tregs, MDSCs, effector memory T cells) and effector cells (CD4+ T cells, CD8+ T cells, gamma-delta T cells, NK cells, central memory T cells), in patients with high-risk thyroid cancer would be different from those in low-risk thyroid cancer. Hence, we aimed to identify immunophenotypes associated with follicular cell-derived thyroid cancer prognosis to recognize patients with aggressive thyroid cancer that could benefit from personalized management including novel immunotherapies.

## Materials and methods

### Study population and patient samples

We performed an institutional review board–approved pilot prospective cohort study of 32 adults with follicular cell-derived thyroid cancer who underwent initial or subsequent surgical management at a tertiary care cancer center. Informed consent was obtained from each participant and the research was completed in accordance with the Declaration of Helsinki as revised in 2013. We excluded patients with medullary thyroid cancer. Peripheral blood was collected at the time of surgery in tubes containing K_2_EDTA anticoagulant. The electronic medical record was utilized to gather clinical, radiographic, laboratory, and pathologic data.

### Outcomes

Participants were categorized into low, intermediate, and high risk for recurrence groups according to the 2015 revised American Thyroid Association (ATA) guidelines (26); into tumor node metastasis (TNM) stage 1, 2, 3 or 4 according to the 8^th^ American Joint Committee on Cancer (AJCC) edition (27); and according to the presence or absence of loco-regional and distant recurrence or progression during follow-up. Circulating immunophenotypes were compared among each of these groups.

### Flow cytometry of peripheral blood

**Figure 1:**
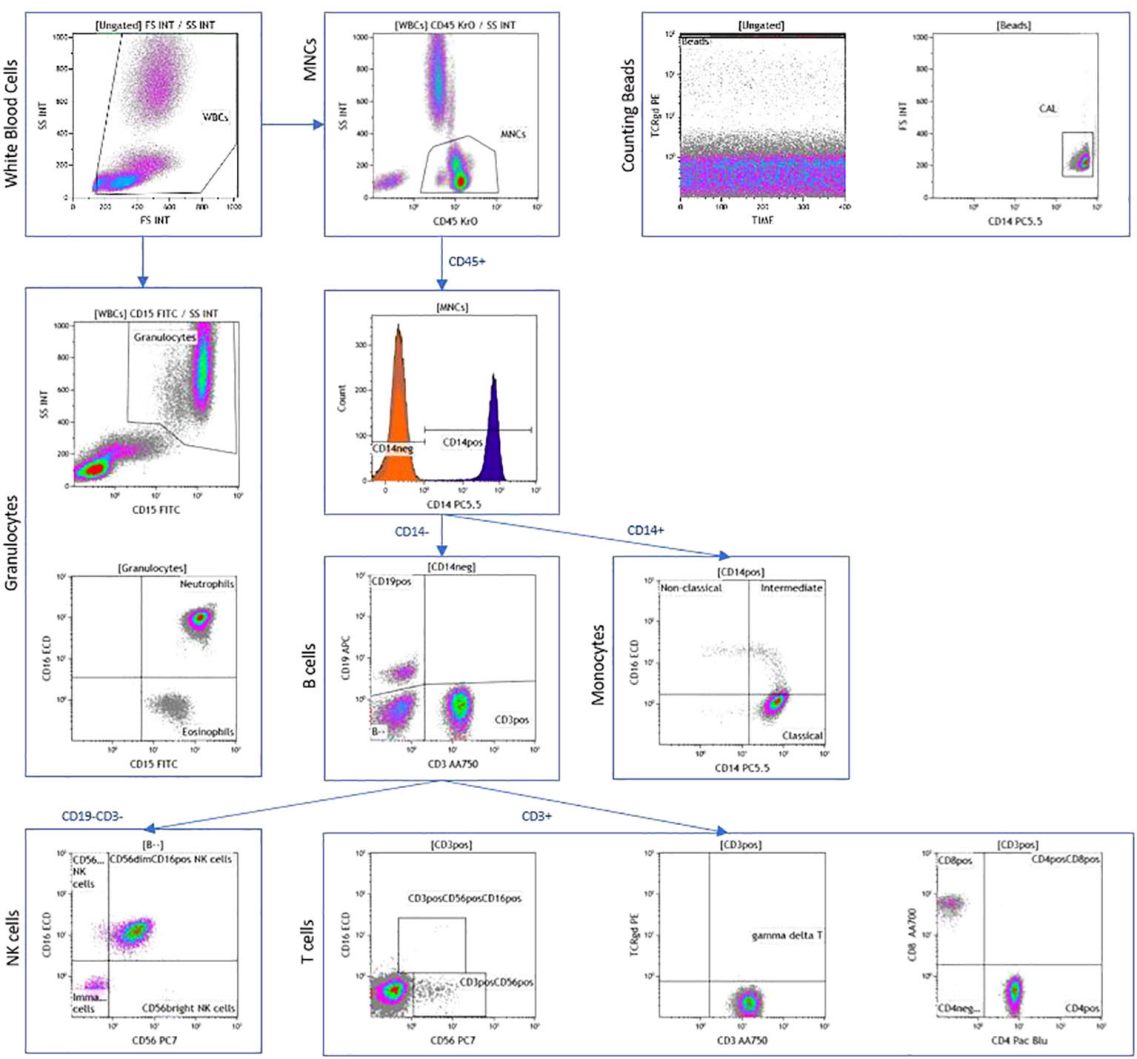
Peripheral blood immunophenotyping via flow cytometry demonstrating dot plots in a patient with AJCC TNM stage 4 and ATA high-risk follicular cell-derived thyroid cancer.

This was performed using a 10-color flow cytometer panel of antibodies for quantification of all major leukocyte populations (**Supplementary Table 1**) as previously published (22–25). Samples were run on the Beckman Coulter Gallios 3-laser, 10-color flow cytometer that was calibrated per the manufacturer’s recommendations each day of use. List mode data (LMD) files were analyzed using Kaluza software version 1.2. Leukocyte populations of interest were colored by the representative gate or “backgated” using histograms of selected stained cell populations. Kaluza software was used to create radar plots. Leukocytes were quantified as: Cell count/microL = count (“Phenotype”) X (Flow-Count Flourospheres/microL)/count. This allowed quantification of the absolute number and percentage of immune cells. The gating strategy is demonstrated in **Figure 1**.

### Statistical analyses

Descriptive statistics were used to determine mean and standard deviation or median and range for continuous variables, and number and percentage for categorical variables. Comparisons of immune cells between different cohorts were evaluated for statistical significance via the student t-test. A p-value <0.05 was used to classify statistically significant, and a p-value <0.001 to classify highly statistically significant differences. All graphical representations and statistical analyses were performed in Prism 9.4.0 (GraphPad, San Diego, CA).

## Results

### Characteristics of thyroid cancer patients

In this cohort of 32 adult follicular cell-derived thyroid cancer patients followed for a median of 57.7 months from initial thyroid cancer surgery, the median age was 58 years (range 32, 85), all were non-Hispanic Caucasian, and 47% were females. Thyroid cancer was well-differentiated in 20 (62%), poorly differentiated in 5 (16%) and anaplastic in 7 (22%). The most frequent AJCC stage was 4 (59%) and ATA risk of recurrence category was high in 18 (56%). Radioactive iodine (RAI) treatment was provided to 17 patients, and tyrosine kinase inhibitor (TKI) to 6 patients after the initial thyroid cancer surgery and blood draw for flow cytometry. The median overall survival was 20 years. The mortality rate was 19%, of which four had distant metastasis at presentation while the other two had distant spread during follow-up (**Table 1**). Due to few patients with ATA low risk cancer (n=4), we combined those with ATA low and intermediate risk when comparing immunophenotypes to high ATA risk group.

**Table 1:** Demographic and clinical characteristics of 32 adult patients with follicular cell-derived thyroid cancer that underwent immunophenotyping by peripheral blood flow cytometry.

Characteristics (median, range or n, %)	Total sample = 32
Age, years	58.5 (32, 85)
Females	15 (46.9)
Caucasian race	32 (100)
Type of thyroid carcinoma Papillary Classic Oncocytic variant Follicular variant Follicular Hurthle cell Poorly differentiated Anaplastic	11 (34.4) 1 (3.1) 2 (6.2) 3 (9.4) 3 (9.4) 5 (15.6) 7 (21.9)
Autoimmune thyroid disease Hashimoto’s (positive TPOAb or chronic lymphocytic thyroiditis on pathology) Graves’ (positive TRAb)	4 (12.5) 2 (6.2)
AJCC TNM stage* 1 2 3 4a 4b 4c	6 (18.7) 1 (3.1) 6 (18.7) 6 (18.7) 3 (9.4) 10 (31.2)
T category* T1a T1b T2 T3 T4a T4b	1 (3.1) 5 (15.6) 3 (9.4) 13 (40.6) 4 (12.5) 6 (18.7)
N category N0 N1a N1b	3 (9.4) 6 (18.7) 23 (71.9)
M status M0 M1	19 (59.4) 14 (40.6)
ATA initial risk stratification Low Intermediate High	4 (12.5) 10 (31.2) 18 (56.3)
Radioactive iodine therapy	17 (53.1)^*^
Tyrosine kinase inhibitor therapy	6 (18.7) (n=4 also received RAI)
Duration from thyroidectomy to last follow-up or death (months)	57.7 (2, 491.8)
Disease status during follow-up Distant spread Loco-regional stable No evidence of disease	21 (65.6) 4 (12.5) 7 (21.9)
Mortality	6 (18.7)^#^
Median overall survival (months)	241.9

*n=8 ATA intermediate-risk and n=9 high-risk; n=11 M0 and n=6 M1; ^#^all had distant spread and were ATA high-risk, n=4 of which also had M1 at surgery, all were AJCC TNM stage 4.

### Circulating immunophenotype comparisons by AJCC stage of thyroid cancer

**Figure 2:**
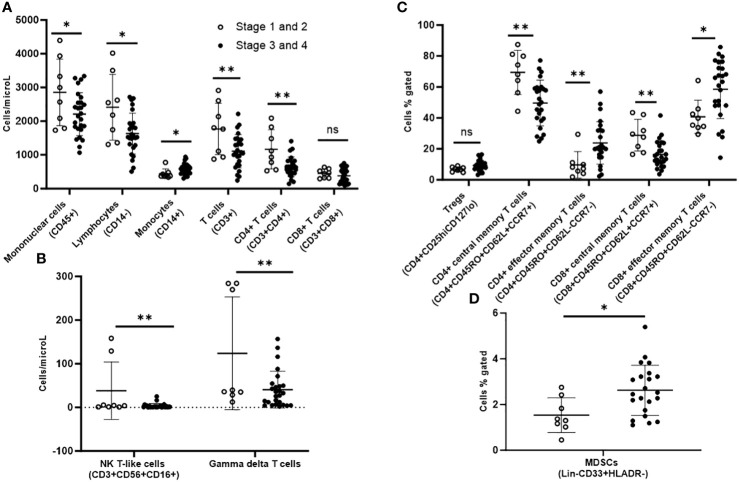
Peripheral blood immunophenotyping via flow cytometry comparing patients with AJCC TNM stages 3 and 4 versus stages 1 and 2 follicular cell-derived thyroid cancer. **(A)** shows immunophenotyping in the T cell, B cell and NK cell panel. **(B)** shows the NK T-like and gamma delta subpopulations of T cells. **(C)** shows further subtyping of T cells to characterize T regs and memory T cells. **(D)** shows MDSCs. *p<0.05; **p<0.01. NK cell: natural killer cell; Treg: T regulatory cell; MDSC: myeloid-derived suppressor cell.

On immunophenotyping in the T cell, B cell, and NK cell panel, patients with AJCC stage 3/4 demonstrated overall fewer circulating mononuclear cells (CD45+) as compared to stage 1/2 (2210 vs. 2855 cells/microL; p=0.04). They also had more monocytes (CD14+) (579 cells/microL vs. 442 cells/microL; p=0.04) but fewer total lymphocytes (CD14-) (1632 cells/microL vs. 2413 cells/microL; p=0.01). Within the lymphocyte compartment, differences in lymphocyte populations were specific to the T cell compartment. Patients with stage 3/4 demonstrated fewer T cells (CD3+) (1111 cells/microL vs. 1770 cells/microL; p=0.007). In sub-populations of T cells, they exhibited fewer CD4+ T cells (646 cells/microL vs. 1165 cells/microL; p=0.002) gamma-delta T cells (40.8 cells/microL vs. 124 cells/microL; p=0.007) and natural killer (NK) T-like cells (CD3+CD56+) (3.43 cells/microL vs. 38.2 cells/microL; p=0.009), but there were no differences in CD8+ T cells or NK cells when compared to stage 1/2 (**Figures 2A, B**).

Upon further subtyping of T cells, there was a trend towards more Tregs (CD4+CD25hiCD127lo) among patients with AJCC stage 3/4 as compared to stage 1/2, but the difference was not statistically significant (p=0.06). We observed more circulating CD4+ effector memory T cells (CD4+CD45RO+CD62L-CCR7-) (23.9% vs. 9.7%; p=0.009) and CD8+ effector memory T cells (CD8+CD45RO+CD62L-CCR7-) (58.5% vs. 40.7%; p=0.02), but fewer CD4+ central memory T cells (CD4+CD45RO+CD62L+CCR7+) (49.6 vs. 69.5; p=0.002) and CD8+ central memory T cells (CD8+CD45RO+CD62L+CCR7+) in patients with stage 3/4 versus stage 1/2 disease (**Figure 2C**).

Stage 3/4 thyroid cancer patients also had more circulating myeloid-derived suppressor cells (MDSCs; Lin-CD33+HLADR-) as subset of mononuclear cells (2.63% vs. 1.54%; p=0.02) compared with stage1/2 (**Figure 2D**).

### Circulating immunophenotype comparisons by ATA risk stratification of thyroid cancer

**Figure 3:**
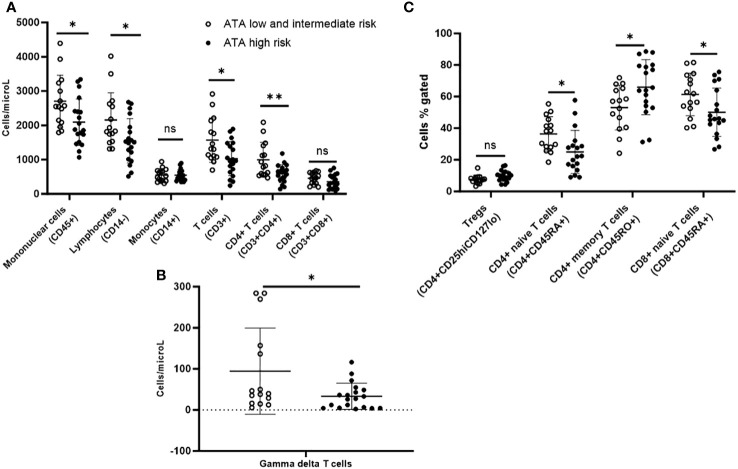
Peripheral blood immunophenotyping via flow cytometry comparing patients with ATA high-risk versus low/intermediate-risk of recurrence follicular cell-derived thyroid cancer. **(A)** shows immunophenotyping in the T cell, B cell and NK cell panel. **(B)** shows gamma delta subpopulation of T cells. **(C)** shows further subtyping of T cells to characterize T regs and memory T cells. *p<0.05; **p<0.01. NK cell: natural killer cell; Treg: T regulatory cell.

On immunophenotyping in the T cell, B cell, and NK cell panel, patients with ATA high-risk thyroid cancer demonstrated overall fewer circulating mononuclear cells (CD45+) compared with ATA low/intermediate risk. They also had fewer total lymphocytes, and within the lymphocyte compartment, fewer T cells (CD3+) but no difference in B cells (CD19+). They also had fewer CD4+ T cells and gamma-delta T cells, but there were no differences in CD8+ T cells or NK cells compared with ATA low/intermediate-risk (**Figures 3A, B**).

Upon further subtyping of T cells, there was a trend towards more Tregs (CD4+CD25hiCD127lo) among patients with ATA high compared to ATA low/intermediate risk, but the difference was not statistically significant (p=0.06). We observed more circulating CD4+ memory T cells (CD4+CD45RO+) (65.9% vs. 53.1%; p=0.03) and a non-significant trend for more CD8+ memory T cells (CD8+CD45RO+) (45.5% vs. 36.1%; p=0.06), but fewer CD4+ naive T cells (CD4+CD45RA+) (25% vs. 36.4%; p=0.014) and CD8+ naive T cells (CD8+CD45RA+) (50.1% vs. 61.4%; p=0.03) in ATA high-risk compared with ATA low/intermediate-risk patients (**Figure 3C**). There was a trend towards more MDSCs in ATA high-risk patients, but the difference was not statistically significant.

### Circulating immunophenotype comparisons by course of thyroid cancer

**Figure 4:**
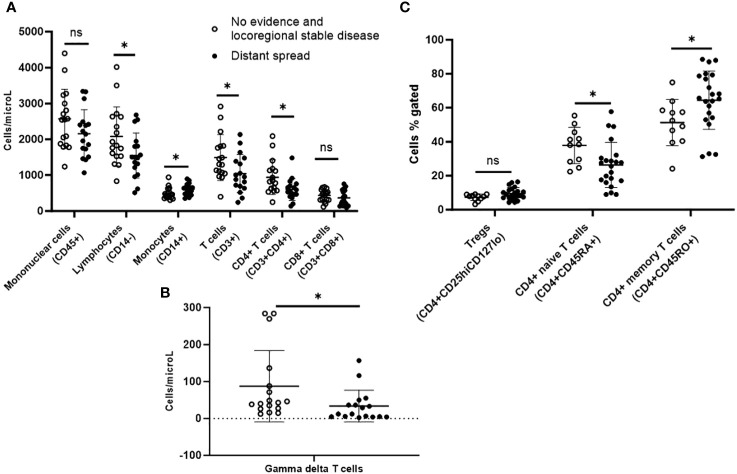
Peripheral blood immunophenotyping via flow cytometry comparing patients with distant metastases versus no recurrence or only loco-regional disease during follow-up follicular cell-derived thyroid cancer. *p<0.05; **p<0.01. **(A)** shows immunophenotyping in the T cell, B cell and NK cell panel. **(B)** gamma delta subpopulation of T cells. **(C)** shows further subtyping of T cells to characterize T regs and memory T cells. *p<0.05; **p<0.01. NK cell: natural killer cell; Treg: T regulatory cell.

During median follow-up of 57.7 years since initial thyroid cancer surgery, 21 patients demonstrated distant spread, of which 14 patients already had distant metastases during initial presentation. Due to the small sample of 6 patients that developed new distant metastases during follow-up, we compared 21 patients with any distant spread to 11 patients who had no evidence of disease or locoregional stable disease during this follow-up duration (**Table 1**). On immunophenotyping in the T cell, B cell, and NK cell panel, patients who demonstrated distant metastases during their disease course demonstrated overall fewer circulating mononuclear cells (CD45+) compared to those who had no evidence of disease or locoregional stable disease. They also had fewer total lymphocytes, and within the lymphocyte compartment, fewer T cells (CD3+) but no difference in B cells (CD19+). They had fewer CD4+ T cells and gamma-delta T cells, but there were no differences in CD8+ T cells or NK cells when compared to those with no evidence of disease or locoregional stable disease (**Figures 4A, B**).

Upon further subtyping of T cells, there was a trend towards more Tregs (CD4+CD25hiCD127lo) among patients who developed distant metastases during their disease course compared with those who had no evidence of disease or locoregional stable disease, but the difference was not statistically significant (p=0.06). We also observed more circulating CD4+ memory T cells (CD4+CD45RO+) (64.4% vs. 51.3%; p=0.03) but fewer CD4+ naive T cells (CD4+CD45RA+) (26.3% vs. 37.9%; p=0.02) in patients who developed distant metastases during their disease course compared with those who had no evidence of disease or locoregional stable disease (**Figure 4C**). There was a trend towards more MDSCs in these patients, but the difference was not statistically significant.

### Additional circulating immunophenotype comparisons

There were no significant differences in the circulating immunophenotypes between patients treated when comparing patients based on age (55 years as cut-off), sex (male versus female), RAI treatment status (17 received versus the rest), TKI treatment status (6 received versus the rest). However, our sample of 32 subjects is not large enough to provide adequately powered conclusions from these negative results.

## Discussion

In this study of 32 adults with follicular cell-derived thyroid cancer, we demonstrated that circulating immunophenotypes serve as prognostic biomarkers. Overall, aggressive thyroid cancer at presentation or during follow-up was characterized by more immune suppressor cells (MDSCs and trend for Tregs) but fewer immune effector cells (CD4+ T cells, gamma-delta T cells and NK T-like cells) and altered memory T cell subtypes compared to less aggressive thyroid cancer. The immunophenotypes were not different based on sex or age of the patients. These findings prove our hypothesis that suppressor circulating immunophenotypes and altered T cell signaling portend a worse thyroid cancer prognosis.

Upon initial analysis, there was a down-regulation of T cell populations specifically in the CD4+ T cell compartment in patients with aggressive thyroid cancer defined as: i) AJCC TNM stage 3 or 4, ii) high risk of recurrence by ATA criteria, or iii) demonstrating distant metastases during follow-up. Available literature evaluating the presence of chronic lymphocytic thyroiditis in the tumor microenvironment without further identifying the specific cell types has demonstrated contradictory results in terms of its association with prognosis (11–14). Hence, we performed a more in-depth analysis of subsets of T cells and NK cells. There are conflicting data regarding the role of CD8+ cytotoxic T cells with one study demonstrating good (20) while another poor prognosis (21). In our study, the lower ratio of CD4/CD8, with significantly fewer CD4+ T cells but no significant difference in CD8+ T cells in patients with more aggressive disease supports the role of CD4+ cytotoxic and helper T cells in mediating immune response against cancer cells.

The lower number of circulating gamma-delta T cells in those with more aggressive thyroid cancer has not been reported in previous thyroid cancer studies of either the tumor microenvironment or peripheral blood. T lymphocytes expressing the gamma-delta form of the T-cell receptor are a distinct functional class whose physiologic role is not completely understood. Their activation results in cell proliferation, proinflammatory cytokine and chemokine secretion, and alteration of cell surface phenotypes (28). It has been postulated that they contribute more to immunoregulation and tissue repair than to immunoprotection (29). They contribute to the pathogenesis of autoimmune disorders, including autoimmune (Hashimoto’s) thyroiditis (30). In addition, they have demonstrated an anti-tumor role via their antigen-presenting cell-like effects in gastric cancer (31) but to our knowledge, have not been evaluated in thyroid cancer. This significant finding of our study supports the hypothesis that the immune suppressor phenotype is associated with thyroid cancer aggressiveness. These cells should be investigated for avenues of immune upregulation.

Another important focus of our study was to identify MDSCs, which were significantly higher in those with stage 3 and 4 thyroid cancer and there was also a similar but non-significant trend in those with ATA high-risk and distant metastases on follow-up. MDSCs are a heterogeneous cell population that suppresses T cell and NK cell function. They arise from myeloid progenitor cells that do not differentiate into mature dendritic cells, granulocytes, or macrophages (32–34). They play a major role in immune evasion and tumor progression, however, a clear consensus on which phenotypes are most relevant in cancer patients has not been reached (34). Studies have demonstrated their immunosuppressive role in cancers including squamous head and neck cancer (35), breast cancer, and non-small cell lung cancer (36). We identified these cells as CD33+ and lineage negative (Lin-) meaning CD3-, CD14-, CD19-, and CD57- like previous studies (36). To our knowledge, only one study has evaluated circulating MDSCs in thyroid cancer patients demonstrating their association with clinicopathologically advanced thyroid cancer (16). Our results validate these findings suggesting that MDSCs are novel biomarkers for predicting aggressiveness of thyroid cancer at diagnosis and should be investigated as therapeutic targets in advanced thyroid cancer.

Tregs (CD4+CD25hiCD127lo) inhibit the anti-tumor response by producing IL-10 and expressing immune checkpoints CTLA-4 and PD-1, hence higher number of these cells in the circulation is associated with more aggressive thyroid cancer (15). The same study showed that PD-1+ T cells (15) were also elevated in more aggressive thyroid cancer. In our study, there was a trend towards more Tregs among patients with AJCC stage 3/4, ATA high-risk, and those that developed distant metastases, but the difference was not statistically significant. Due to the limited sample size, we cannot conclude if Tregs are associated with aggressive thyroid cancer or not. However, our findings support further investigation of their role in a larger cohort of thyroid cancer patients because they could be a potential target for immunotherapy that functions in an antagonistic manner.

In the literature, certain immunoregulatory subtypes of NK cells (CD3-CD16-CD56+)are reported to be associated with pathologically aggressive thyroid cancer (37), however, the main immune effector subtype of NK cells have been shown to clear cancerous cells with low MHC expression. We did not find significant differences in NK cells between the thyroid cancer subgroups; however, this could be a limitation of our sample size and characteristics. The NK T-like cells (CD3+CD56+) combine the characteristics of T (CD3+) and NK (CD56+) cells. The exact pathophysiological role of these cells remains unknown although literature has reported on their effector role in autoimmune diseases (38), and cytotoxic role against infectious diseases (39) and cancer (40, 41). They are reduced in circulation in patients with metastatic as compared to non-metastatic colorectal cancer (42) but have not been investigated in thyroid cancer. Their ability to clear cancerous cells with low MHC expression supports our investigation of their role in thyroid cancer. In our study, NK T-like cells were reduced in patients with advanced clinicopathologic thyroid cancer suggesting their cytotoxic role against tumor cells. Future studies should investigate ways for upregulating these cells as a novel form of therapy against advanced cancer.

In our study, advanced stage (III/IV) thyroid cancer at presentation was characterized by more effector memory T cells but fewer central memory T cells. These differences in subtypes of memory T cells were not significant when comparing by ATA risk or course during follow-up, but in general, there were more memory T cells and fewer naïve T cells in ATA high risk compared to low/intermediate risk and among patients who developed distant metastases during their disease course compared with those who had no evidence of disease or locoregional stable disease. Since central memory T cells express the chemokine receptor CCR7, they traffic to lymph nodes and interact with dendritic cells. Effector memory T cells lacking CCR7 expression migrate to areas of inflamed tissue and display immediate effector function (43, 44). Studies have shown that in chronic infectious processes, there is a gradual shift in the composition of the memory T cell pool from an effector to a central memory phenotype (45, 46). Effector memory cells present an immediate, but not sustained, defense at pathogen sites of entry, whereas central memory T cells sustain the response by proliferating in the secondary lymphoid organs and producing a supply of new effectors (47–49) In addition, effector memory T cells are less efficient than central memory T cells at mediating recall responses in terms of proliferation and accumulation at inflammatory sites (46, 50). Hence, even though not definitively certain, the differences in these memory T cells observed in our study are consistent with the immune suppressor phenotype observed in aggressive thyroid cancer. CCR7 expression has been shown to be lower in poorly differentiated compared with differentiated thyroid cancer (51) which fits with more effector memory T cells (less CCR7) in advanced stages of thyroid cancer in our study. While previous studies have evaluated pathological aggressiveness, our study is novel in investigating this in a cohort predominantly comprised of differentiated thyroid cancer patients with adequate follow-up. Our results suggest that T cell trafficking is altered in advanced stages of thyroid cancer, thus shedding light on both the biology and potentially prognostic applications.

Our study is limited by its small sample size which precludes comparing immunophenotype differences between several types of follicular cell-derived thyroid cancer, stage 3 and 4 patients, and ATA intermediate and high-risk patients. The overall few events of distant progression do not allow us to evaluate the relationship between time to cancer progression and various immune phenotypes in this study. All patients being non-Hispanic Caucasian prevented us from analyzing differences by race or ethnicity. Additionally, the lack of significant differences in circulating immunophenotypes based on treatment with RAI or TKI, and lack of significant differences in Tregs or NK cells between various clinicopathologic subgroups could be due to inadequately powered sample size, hence should not be inferred as definitive lack of difference. Importantly, circulating immune phenotypes may not reflect the *in-situ* tumor microenvironment, including spatial relationships among immune cells. Tumor-associated macrophages are associated with poor outcomes such as lymph node metastases (52), larger tumor size (53) and reduced survival (54, 55), but are not enough in circulation to perform comparisons, hence these and other tumor-infiltrating immune cells should be the focus of further research on tumor and tumor-adjacent tissue to characterize the thyroid cancer tumor microenvironment. Also, we did not perform an investigation of intracellular factors or functionality of immune cells in our study, did not serially analyze the immunophenotypes over the course of disease or compare them to inflammatory thyroid diseases. The heterogeneity in our results and the overlap in immunophenotypes between the compared groups despite significant differences are also limitations. Hence, future studies with larger cohorts of thyroid cancer patients are required to investigate these important factors in thyroid cancer prognostication. Our pilot study’s strengths include its prospective nature, comprehensiveness of immunophenotyping, evaluation of clinically important outcomes, being the first to demonstrate fewer gamma-delta T cells and NK T-like cells, and only the second to demonstrate more circulating MDSCs amongst patients with advanced thyroid cancer. These findings provide a strong basis for further investigation into the immune phenotypes in circulation and tumor microenvironment in a larger cohort of patients with thyroid cancer.

In conclusion, we have demonstrated that aggressive follicular cell-derived thyroid cancer either at presentation or during the disease course is associated with circulating suppressor immunophenotypes characterized by fewer CD4+ T cells, gamma-delta T cells, and NK T-like cells but more MDSCs; and altered memory T cell subtypes. These immunophenotypes serve as prognostic biomarkers for advanced thyroid cancer. Future studies with larger cohorts should evaluate the changes in circulating and tumor-infiltrating immunophenotypes with thyroid cancer progression and investigate the role of immunotherapies antagonistic to MDSCs and Tregs while upregulating NK T-like and gamma-delta T cells as well as influencing T cell signaling in advanced thyroid cancer.

## Data availability statement

The original contributions presented in the study are included in the article/**Supplementary Material**. Further inquiries can be directed to the corresponding author.

## Ethics statement

The studies involving humans were approved by Mayo Clinic Institutional Review Board. The studies were conducted in accordance with the local legislation and institutional requirements. The participants provided their written informed consent to participate in this study.

## Author contributions

AK: Data curation, Formal analysis, Investigation, Methodology, Validation, Visualization, Writing – original draft, Writing – review & editing. MG: Data curation, Formal analysis, Investigation, Methodology, Resources, Software, Validation, Visualization, Writing – review & editing. SB: Formal analysis, Investigation, Methodology, Project administration, Validation, Visualization, Writing – review & editing. AD: Conceptualization, Methodology, Resources, Supervision, Validation, Writing – review & editing. DD: Data curation, Methodology, Visualization, Writing – review & editing. MR: Conceptualization, Data curation, Formal analysis, Funding acquisition, Investigation, Methodology, Project administration, Resources, Software, Supervision, Validation, Visualization, Writing – original draft, Writing – review & editing.
