# Supplementary material for: Circulating immunophenotypes are potentially prognostic in follicular cell-derived thyroid cancer
Source: Front Immunol. 2024 Jan 3;14:1325343. doi: 10.3389/fimmu.2023.1325343 (PMC10792034; doi:10.3389/fimmu.2023.1325343)
Supplement: Supplementary file 1 [file Table_1.doc]

Supplemental table 1. Antibodies and reagents used for flow cytometry.

| Antibody | Company | Catalog number | Clone number |
| --- | --- | --- | --- |
| CCR3 APC | R&D Systems | FAB155A | 61828 |
| CCR7 FITC | R&D Systems | FAB197F | 150503 |
| CD2 PE | Coulter | IM0443U | 39C1.5 |
| CD3 APC-750 | Coulter | A66329 | UCHT1 |
| CD3 KrO | Coulter | B00068 | UCHT1 |
| CD3 PC7 | Coulter | 6607100 | UCHT1 |
| CD4 Pac Blu | Coulter | A82789 | 13B8.2 |
| CD5 AA750 | Coulter | A78836 | BL1a |
| CD5 PC7 | Coulter | A51075 | BL1a |
| CD7 APC | Coulter | A97050 | 8H8.1 |
| CD8 FITC | Coulter | IM0451U | B9.11 |
| CD8 APC-700 | Coulter | A66332 | B9.11 |
| CD8 KrO | Coulter | B00067 | B9.11 |
| CD11b PC7 | Coulter | A54822 | Bear1 |
| CD11c PC5 | Coulter | B19719 | BU15 |
| CD14 ECD | Coulter | IM2707U | RMO52 |
| CD14 PC5.5 | Coulter | A70204 | RMO52 |
| CD15 FITC | Coulter | IM1423U | 80H5 |
| CD15 Pac Blu | Coulter | A74775 | 80H5 |
| CD16 APC-750 | Coulter | A66330 | 3G8 |
| CD16 APC-700 | Coulter | B20023 | 3G8 |
| CD16 ECD | Coulter | A33098 | 3G8 |
| CD19 APC | Coulter | IM2470U | J3-119 |
| CD19 APC-700 | Coulter | A78837 | J3-119 |
| CD20 ECD | Coulter | IM3607U | B9E9 |
| CD21 Pac Blu | Coulter | B09982 | BL13 |
| CD24 APC-750 | Coulter | B10738 | ALB9 |
| CD25 PC5.5 | Coulter | A79386 | B1.49.9 |
| CD27 PE | Coulter | IM2578 | 1A4CD27 |
| CD28 APC-750 | Coulter | B08757 | CD28.2 |
| CD32 eFluor710 | eBioscience | 46-0392-42 | 6C4 |
| CD33 APC | Coulter | IM2471U | D3HL60.251 |
| CD38 PC5.5 | Coulter | A70205 | LS198-4-3 |
| CD40 APC | BD | 555591 | 5C3 |
| CD44 PerCp Cy 5.5 | BD | 560531 | G44-26 |
| CD45 KrO | Coulter | A96416 | J.33 |
| CD45RA APC-750 | Coulter | A86050 | 2H4LDH11LDB9 |
| CD45RO ECD | Coulter | IM2712U | UCHL1 |
| CD49d AA750 | Coulter | B16893 | HP2/1 |
| CD56 PC7 | Coulter | A51078 | N901 (HLDA6) |
| CD62L APC | BD | 559722 | DREG-56 |
| CD63 PE | Coulter | IM1914U | CLBGran/12 |
| CD64 PC7 | Coulter | B06025 | 22 |
| CD66b FITC | Coulter | IM0531U | 80H3 |
| CD66b APC-750 | Coulter | B08756 | 80H3 |
| CD80 FITC | Coulter | IM1853U | MAB104 |
| CD86 APC | BD | 555660 | 2331 (FUN-1) |
| CD123 PE | Coulter | A32535 | SSDCLY107D2 |
| CD127 AA700 | Coulter | A71116 | R34.34 |
| Gamma delta TCR PE | Coulter | IM1418U | IMMU510 |
| HLA-DR ECD | Coulter | IM3636 | Immu-357 |
| HLA-DR Pac Blu | Coulter | A74781 | Immu-357 |
| IgD FITC | Coulter | B30652 | IA6-2 |
| IgM APC | Coulter | 735972 | SA-DA4 |
| Lineage-2 FITC | BD | 643397 | SK7  SJ25C1  L27  NCAM16.2 |
